# Selection of reliable reference genes for quantitative RT-PCR in garlic under salt stress

**DOI:** 10.7717/peerj.7319

**Published:** 2019-07-16

**Authors:** Guanglong Wang, Chang Tian, Yunpeng Wang, Faxiang Wan, Laibao Hu, Aisheng Xiong, Jie Tian

**Affiliations:** 1 School of Life Science and Food Engineering, Huaiyin Institute of Technology, Huaian, Jiangsu, China; 2 Key Laboratory of Landscape Agriculture, Ministry of Agriculture, College of Horticulture, Nanjing Agricultural University, Nanjing, Jiangsu, China; 3 State Key Laboratory of Crop Genetics and Germplasm Enhancement, College of Horticulture, Nanjing Agricultural University, Nanjing, Jiangsu, China; 4 Academy of Agriculture and Forestry Sciences of Qinghai University (Qinghai Academy of Agriculture and Forestry Sciences), Qinghai Key Laboratory of Vegetable Genetics and Physiology, State Key Laboratory of Plateau Ecology and Agriculture, Qinghai University, Qinghai, China

**Keywords:** Reference genes, Garlic, Salt stress, qRT-PCR, Gene expression

## Abstract

Quantitative real-time reverse-transcriptase PCR (qRT-PCR) has been frequently used for detecting gene expression. To obtain reliable results, selection of suitable reference genes is a fundamental and necessary step. Garlic (*Allium sativum*), a member from Alliaceae family, has been used both as a food flavoring and as a traditional medicine. In the present study, garlic plants were exposed to salt stress (200 mM NaCl) for 0, 1, 4 and 12 h, and garlic roots, bulbs, and leaves were harvested for subsequent analysis. The expression stability of eight candidate reference genes, *eukaryotic translation initiation factor* 4*α* (*eIF-4α*), *actin* (*ACTIN*), *tubulin β*-7 (*TUB7*), *TAP42-interacting protein of 41 kDa* (*TIP41*), *glyceraldehyde-3-phosphate dehydrogenase* (*GAPDH*), *SAND family protein* (*SAND*), *elongation factor 1 alpha* (*EF*-1*α*), and *protein phosphatase 2A* (*PP2A*) were evaluated by geNorm, NormFinder, and BestKeeper. All genes tested displayed variable expression profiles under salt stress. In the leaf and root group, *ACTIN* was the best reference gene for normalizing gene expression. In garlic clove, *ACTIN* and *SAND* were the least variable, and were suitable for gene expression studies under salt stress; these two genes also performed well in all samples tested. Based on our results, we recommend that it is essential to use specific reference genes in different situations to obtain accurate results. Using a combination of multiple stable reference genes, such as *ACTIN* and *SAND*, to normalize gene expression is encouraged. The results from the study will be beneficial for accurate determination of gene expression in garlic and other plants.

## Introduction

Garlic, one of the most widely cultivated species in the Alliaceae family, has been used by humans for over 4,000 years (Tuan et al., 2011). Although garlic cloves are commonly consumed parts in human diets, other parts, such as garlic scapes and leaves can also be used and eaten (Ruíz-Torres et al., 2017). China makes up a large proportion of garlic production in the world. Owing to abundant sulfur and phenolic compounds, garlic has a wide range of beneficial properties (Jones et al., 2004). Garlic extract was reported to be one of the best antioxidative and disease-preventive foods (Jeong et al., 2016; Wang et al., 2015; Zakarova et al., 2014). Recent studies focused more attention on health-related benefits of garlic; however, there are very few data describing the response of garlic plants to environmental stimuli.

Salt stress is one of the main environmental problems adversely affecting crop growth and production (Nakaminami et al., 2018; Wu et al., 2018; Zhang et al., 2017). The negative impacts of salinity are not only confined to tissue or cellular level but also appear at the genetic and molecular levels (Zhu et al., 2018). Therefore, a proper understanding of plant response to salt stress at the molecular level is of vital importance for developing crops with high productivity under salt stress. Profiling stress-induced differential gene expression is a fundamental and effective way to obtain molecular information.

Due to high specificity and sensitivity, qRT-PCR has become a common choice to detect gene expression patterns under specific conditions (Shakeel et al., 2018). Moreover, it is also utilized for the verification of the high throughput sequencing results (Wang et al., 2018; Zhang et al., 2018). However, the veracity of qRT-PCR can be markedly affected by a battery of factors, such as the condition of the samples, RNA isolation, cDNA synthesis, and the experimental process. Therefore, the internal control that serves as a normalization factor is necessary to minimize the distractions mentioned above.

The perfect internal reference gene ought to be stably expressed in different developmental stages, tissues, and conditions (Wong & Medrano, 2005). Some genes, such as *TUB*, *ACTIN*, and *EF-1α* were stably expressed in some cases, and were usually used for normalization (Kong et al., 2015; Moazzam Jazi et al., 2016). However, recent studies have indicated that no reference gene can be used universally under all situations. That is to say, identification of a reliable reference gene for a specific stage or condition is of vital importance. With the help of different software tools or statistical procedures including BestKeeper (Pfaffl et al., 2004), geNorm (Vandesompele et al., 2002), and NormFinder (Andersen, Jensen & Orntoft, 2004), selection of appropriate reference genes under various conditions has made great progress. To date, selection of appropriate reference genes under various conditions has been reported in a series of plants including celery (Li et al., 2016), Chinese jujube (Bu, Zhao & Liu, 2016), green foxtail (Nguyen, Eamens & Grof, 2018), mulberry (Dai et al., 2018), and kikifruit (Liu et al., 2018). However, there is no report describing the selection of reference genes in garlic under specific conditions.

Salt treatment groups with different time and tissues were used to identify the stable reference genes for normalization. Furthermore, three different types of software including geNorm, NormFinder, and Best-Keeper were applied to detect the variation of the expression of the candidate genes and select the suitable reference genes for normalization of gene expression under given conditions in garlic. The experimental samples completely represent the salt treatments and the introduction of the three statistical algorithms ensures the accuracy of the results.

## Materials & Methods

### Plant material and growth conditions

The experiment was conducted in a glass greenhouse of Huaiyin Institute of Technology. The cloves of garlic cultivar ‘Cangshan siliuban’ were sown in the pots containing vermiculite and organic soil mixture (1:1; v/v). The cloves were allowed to grow for 20 d when the seedlings were about 10 days old. The garlic seedlings were then moved to 1/2 Hogland solution to grow for another 7 days. After this period, the solution was exposed to 200 mM NaCl for 0, 1, 4, and 12 h. Garlic cloves, roots, and leaves were randomly sampled at each time point and stored at −80 °C until further analysis. The experiment was performed with three biological replicates.

### Total RNA isolation and cDNA synthesis

Total RNA was isolated from the frozen samples with a plant RNA extraction kit (Tiangen, Beijing, China) according to the manufacturer’s instructions. RNA concentration was assessed and listed in Table S1. Only the RNA samples with A260/A280 > 1.8 and A260/A230 > 2.0 were used for cDNA synthesis. Prior to cDNA synthesis, genomic DNA contamination was removed using the PrimeScript RT Reagent Kit with gDNA Eraser (TaKaRa, Dalian, China) in accordance with the manufacturer’s instructions. First-strand cDNA synthesis was carried out using the Prime-Script RT reagent Kit (TaKaRa, Dalian, China) according to the manufacturer’s instructions.

### Primer design and qRT-PCR analysis

To guarantee the accuracy of gene expression under salt stress, it is necessary to select and validate the reference genes. Here we obtained the nucleotide sequences of genes that are typically used as reference genes based on garlic transcriptome data (Wang et al., 2019). The eight genes (*eIF-4α*, *ACTIN, TUB7, TIP41, GAPDH, SAND, EF-1α*, *and PP2A*) that have been utilized as internal controls in other plant species (Joseph, Poolakkalody & Shah, 2018), were aligned with the sequences in the garlic transcriptome established by our group (Wang et al., 2019). The nucleotide sequences of candidate reference genes are appended in Table S2. The primer sequences used for qRT-PCR analysis were designed by Primer Premier 6.0 software. The specificity of these primer pairs was checked by gel electrophoresis. The detailed information is displayed in Table 1. The qRT-PCR reactions were carried out in a CFX96 Real-Time PCR Detection System (Biorad, USA). Each 20 µL reaction mixture was comprised of 10 µL of ChamQ SYBR qPCR Master Mix (Vazyme, Nanjing, China), 7.4 µL of deionised water, 2 µL of ten-fold diluted template, and 0.4 µL of each amplification primer. The cycling conditions were set as follows: initial denaturation of 95 °C for 30 s, followed by 40 cycles of denaturation at 95 °C for 5 s, annealing at 60 °C for 30 s. The melting curves were then performed by heating the amplicon from 65 to 95 °C. Negative controls without template were also included at the same time to ensure amplification quality.

**Table 1:** Details of candidate reference genes with primer sequences and amplified characteristics.

Gene name	Primer sequences (forward/reverse)	Amplicon length (bp)	E (%)	*R*^2^
*eIF-4α* (*eukaryotic translation initiation factor* 4*α*)	TTGTGTTGGACGAGGCAGATGAG/ GCAGAGAAGACGCCGACTTGT	106	105.9	0.997
*ACTIN* (*Actin 1* gene)	TGCTCTGGATTATGAACAGGAACTTGA/ CAATCATTGAAGGCTGGAACAACACT	146	103.5	0.999
*TUB7* (*Tubulinβ*-7)	TGGTCGCTACTTGACTGCCTCT/ CACACGCCTGAACATCTCTTGAATG	223	107.0	0.991
*TIP41* (*TAP42-interacting protein of 41 kDa*)	TGGCGGAAGCAAGTGAAGTAGG/ AGAGGTAAGAATAGGACGATTGGAAGA	134	103.5	1.000
*GAPDH* (*Glyceraldehyde-3-phosphate dehydrogenase*)	CTGGATTGAGCCGTGGTTCATCTT/ ATTGCTTGACACCGCCGAAGTT	107	108.1	0.999
*SAND* (*SAND family protein*)	GCGTCAACGAATGTTCCAATTACCA/ TCTCTTCAGTCTCAACTTCATCAGCAT	100	109.4	0.995
*EF-1α* (*Elongation factor 1 alpha*)	CCTGTTCTTGACTGCCACACCTC/ ACCATACCAGCATCGCCATTCTTC	131	103.0	0.990
*PP2A* (*Protein phosphatase 2A*)	GGAGAGTTGGAAGAGGTCTTAGAGG/ TTCAAGCACCGAGCGATCTGTT	92	112.0	0.999

Transcript levels of reference genes were performed in three independent biological samples, each with three technical replicates. The amplification efficiency (*E*, *E* = 10^[−1∕slope]^ − 1) and the correlation coefficients (*R*^2^) for each primer were calculated by utilizing standard curves with ten-fold serial dilutions (10, 10^2^, 10^3^, 10^4^, and 10^5^ dilutions) (Fig. S1).

### Statistical analysis of gene expression stability

To determine the expression variation of the eight candidate reference genes, three software tools, geNorm (https://genorm.cmgg.be/), NormFinder (https://moma.dk/normfinder-software/), and BestKeeper (https://www.gene-quantification.de/bestkeeper.html) were applied.

PCR cycle threshold (Ct) data were detected to quantify the expression of the eight candidate genes in all samples (three tissues, four time points, and three independent biological replicates). The *Ct* values were shown in Table S3 and were directly utilized for BestKeeper analysis, an Excel-based tool using pair-wise correlations. The raw data were also transformed to relative values for geNorm and NormFinder analysis. geNorm software generates a stability measure (M) by excluding the unstable genes using a stepwise method. A lower M value indicates the more stable the expression of the reference gene (Vandesompele et al., 2002). A *M* value below 1.5 has been suggested by Vandesompele and his coworkers (Vandesompele et al., 2002). NormFinder is a MS Excel-based algorithm, which can measure gene expression stability by comparing the intra- and inter-group variation (Andersen, Jensen & Orntoft, 2004). Candidate reference genes with lowest stability values are considered to be the least variable.

### Validation of reference genes

To validate the accuracy of the candidate reference genes, *ascorbate oxidase* (*AO*) were selected from our garlic transcriptome. The primers used for analyzing expression of the genes were listed in Table S4, whereas their digital gene expression in garlic cloves under salt stress was displayed in Fig. S2. The most stable and variable reference genes under salt treatments were validated using the 2^−ΔΔ*Ct*^ method with three biological samples (Livak & Schmittgen, 2001).

## Results

### Assessment of amplification efficiency and specificity

Based on the nucleotide sequences from garlic transcriptome, primer sequences for expression analysis of the eight candidate reference genes (*eIF-4α*, *ACTIN*, *TUB7*, *TIP41*, *GAPDH*, *SAND*, *EF-1α*, and *PP2A*) were designed (Table 1). The amplicon length of the reference genes ranges from 92 to 223 bp. Amplification efficiencies (E) were generated from the standard curves accompanied by great linear relationships (*R*^2^ > 0.99); amplification efficiencies were between 103.0% and 112.0% (Table 1).

### Expression profiling and variation of candidate reference genes

**Figure 1:**
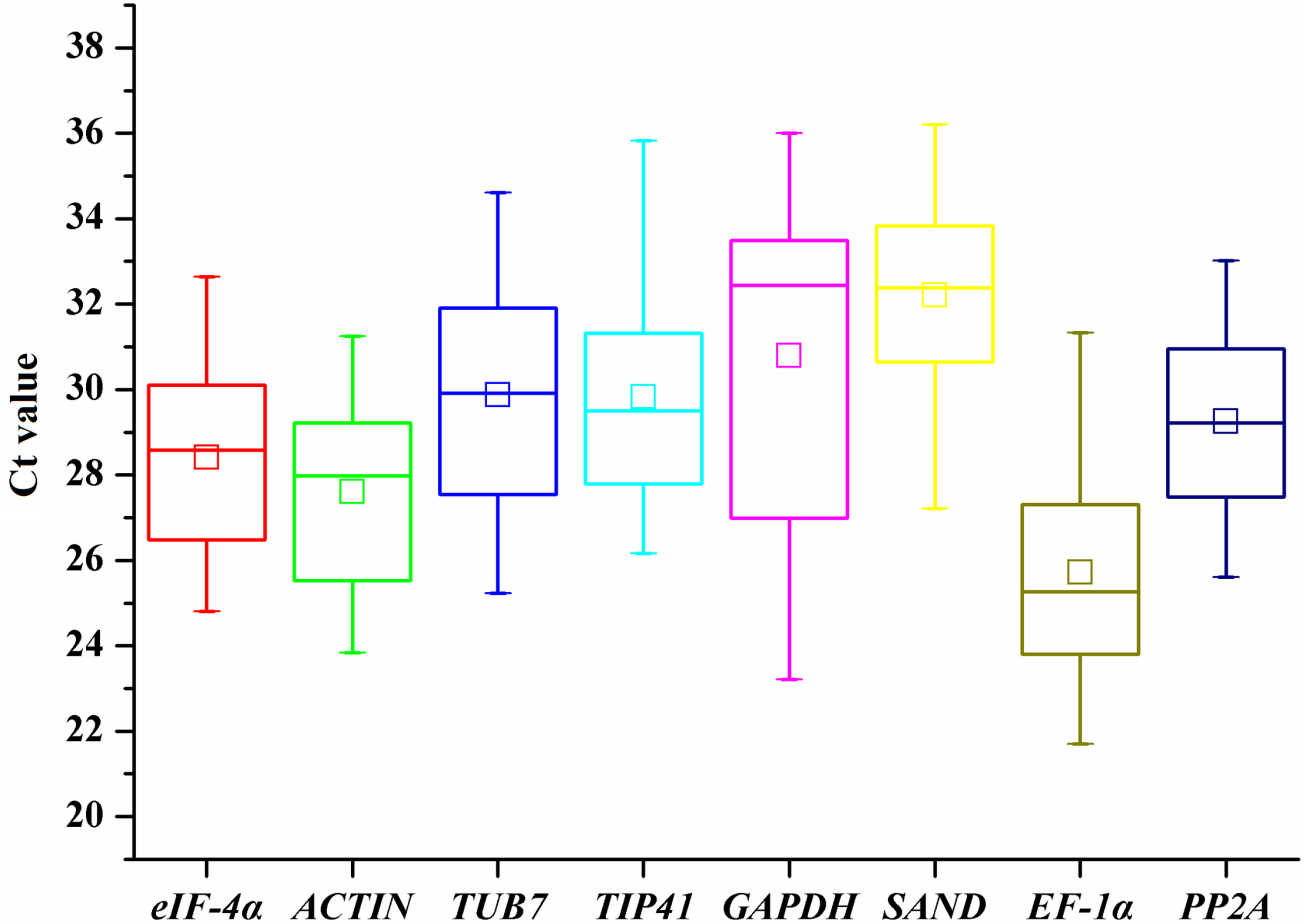
*Ct* values of candidate reference genes in all samples of *A. sativum*. The lines across the outside box represent the median values. The inside boxes indicate the mean values. The 25th and 75th percentiles are depicted by the bottom and top lines of the outside box, respectively. The whiskers indicate the maximum and minimum values.

To provide a general view of the expression of the eight candidate genes, transcript levels of reference genes were expressed as *Ct* values (Fig. 1). The *Ct* values of all the tested samples covered a wide range. The mean *Ct* values of tested genes ranged from 25.72 (*EF-1α*) to 32.21 (*SAND*). The low *Ct* values of *EF-1α* corresponded to high levels of expression, whereas the lowest expression was observed in *SAND* and *GAPDH* genes (Fig. 1). The largest variation in *Ct* values for one gene was detected in *GAPDH* (23.21-36), whereas *ACTIN* (23.84–31.25) and *PP2A* (25.61–33.02) showed relatively moderate variation.

### Statistical analysis of gene expression stability

The expression stability of candidate reference genes in different tissue groups under salt stress was analyzed by using three different software, geNorm, NormFinder, and BestKeeper.

According to the results from geNorm software, most reference genes showed *M* values under the recommended threshold (Table 2). It was observed that the most stable reference genes were not the same in all individual tissue samples. In the clove, *ACTIN* and *SAND* were found to be the most stable reference genes, whereas *EF-1α* and *GAPDH* has exceeded the threshold value. In the leaf, *eIF-4α* and *TUB7* occupied the top two positions among the eight reference genes. By contrast, *SAND* seemed to be the least stable gene. In the root, *TUB7* and *TIP41* were ranked in the first two places and would be reliable for normalizing gene expression. When all tissue samples were taken together, *ACTIN* and *TUB7* were appropriate for normalization under salt stress, and *GAPDH* should be avoided (Table 2).

**Table 2:** Stability comparison of the candidate reference genes analyzed and ranked by geNorm, NormFinder, and BestKeeper.

**Group**	**Rank**	**geNorm**		**NormFinder**		**BestKeeper**		
		**Gene**	**Stability (M)**	**Gene**	**Stability**	**Gene**	**SD**	**CV**
Clove	1	*ACTIN*	081	*PP2A*	0.078	*GAPDH*	0.79	2.34
	2	*SAND*	0.81	*SAND*	0.080	*PP2A*	1.73	5.97
	3	*TUB7*	0.98	*ACTIN*	0.103	*eIF-4α*	1.95	7.01
	4	*TIP41*	1.17	*EF-1a*	0.106	*SAND*	2.16	6.87
	5	*eIF-4α*	1.36	*eIF-4α*	0.116	*ACTIN*	2.17	7.92
	6	*PP2A*	1.49	*TUB7*	0.157	*TIP41*	2.39	8.16
	7	*EF-1a*	1.64	*TIP41*	0.233	*TUB7*	2.50	8.48
	8	*GAPDH*	1.91	*GAPDH*	0.349	*EF-1a*	2.56	9.91
Leaf	1	*eIF-4α*	0.61	*TUB7*	0.060	*PP2A*	1.07	3.53
	2	*TUB7*	0.61	*ACTIN*	0.071	*SAND*	1.12	3.43
	3	*ACTIN*	0.67	*GAPDH*	0.098	*TIP41*	1.36	4.53
	4	*EF-1a*	0.76	*eIF-4α*	0.110	*ACTIN*	1.40	5.01
	5	*TIP41*	0.85	*PP2A*	0.111	*GAPDH*	1.42	5.48
	6	*GAPDH*	0.88	*EF-1a*	0.116	*EF-1a*	1.48	5.81
	7	*PP2A*	0.91	*SAND*	0.120	*eIF-4α*	1.64	5.60
	8	*SAND*	0.94	*TIP41*	0.122	*TUB7*	1.73	5.75
Root	1	*TUB7*	0.75	*TUB7*	0.109	*GAPDH*	0.83	2.53
	2	*TIP41*	0.75	*SAND*	0.116	*eIF-4α*	1.51	5.37
	3	*SAND*	0.79	*TIP41*	0.124	*ACTIN*	1.53	5.57
	4	*ACTIN*	0.88	*ACTIN*	0.128	*PP2A*	1.72	6.04
	5	*eIF-4α*	0.97	*PP2A*	0.129	*EF-1a*	1.80	6.97
	6	*PP2A*	1.04	*EF-1a*	0.135	*SAND*	1.81	5.53
	7	*EF-1a*	1.12	*eIF-4α*	0.137	*TUB7*	1.99	6.66
	8	*GAPDH*	1.35	*GAPDH*	0.337	*TIP41*	2.14	7.07
Total	1	*ACTIN*	0.87	*ACTIN*	0.058	*SAND*	1.71	5.31
	2	*TUB7*	0.87	*EF-1a*	0.072	*ACTIN*	1.75	6.35
	3	*SAND*	0.98	*TUB7*	0.082	*PP2A*	1.76	6.02
	4	*TIP41*	1.07	*SAND*	0.084	*eIF-4α*	1.76	6.20
	5	*eIF-4α*	1.17	*eIF-4α*	0.092	*EF-1a*	1.95	7.58
	6	*EF-1a*	1.28	*PP2A*	0.146	*TIP41*	2.05	6.86
	7	*PP2A*	1.37	*TIP41*	0.167	*TUB7*	2.09	7.00
	8	*GAPDH*	2.11	*GAPDH*	0.274	*GAPDH*	3.23	10.49

According to NormFinder, *TUB7* was the most stable gene in the leaf and root samples, whereas *PP2A* performed well in the clove group. Taken together, *ACTIN* was the best reference gene for normalizing gene expression in all garlic tissues under salinity, while *GAPDH* was the most unstable gene and should be excluded as an internal standard (Table 2).

BestKeeper software was applied for the analysis of standard deviation (SD) and the coefficient of variation (CV) of *Ct* values. The lower the SD and CV values, the higher stability of the reference genes. According to the ranking by BestKeeper (Table 2), the most stable genes were *GAPDH* for clove and root groups, *PP2A* for leaf group, and *SAND* was the most stable gene in all tissue samples.

### Analysis of the optimal number of reference genes for normalization

**Figure 2:**
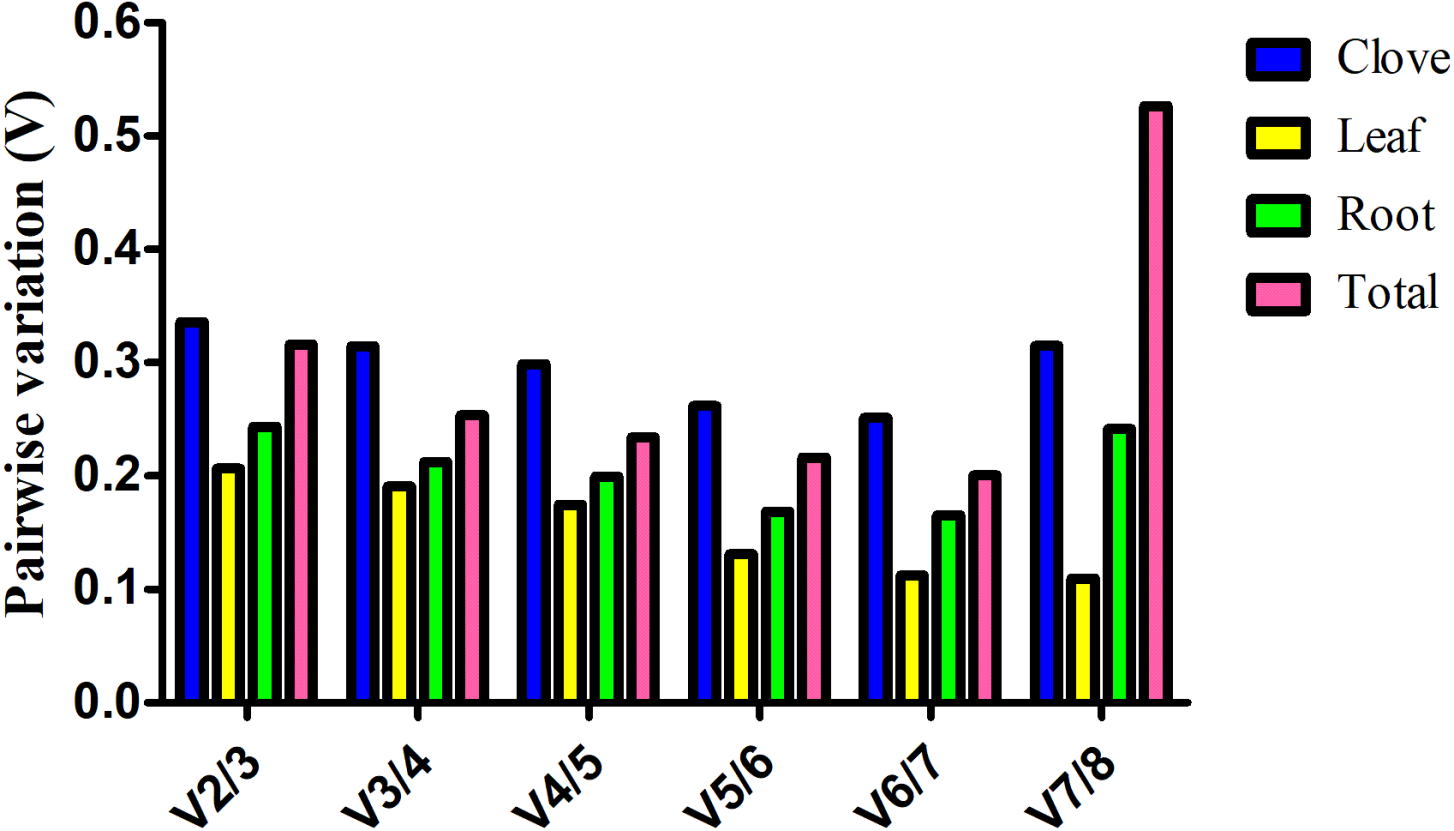
Estimation of the optimal number of reference genes required for normalization of gene expression. Pairwise variation (*Vn*∕*n* + 1) was calculated between two sequential normalization factors *NF*_*n*_ and *NF*_*n*+1_ by geNorm software.

Estimation of the normalization factor (NF), the geometric mean of the expression of combined reference genes, is required for determination of expression of a target gene based on multiple reference genes. geNorm software not only computes NF values, but also provides pairwise variation (*V*), an indicator for determining the optimal number of reference genes for normalizing gene expression. Here, *V* value is always above 0.15 in clove, root, and total samples, suggesting more reference genes are preferred in these groups (Fig. 2). In the leaf group, pairwise variation at the V4/5 value is 0.175, and it falls below the threshold of 0.15 with the addition of next gene, indicating that the proper normalization requires at least five reference genes (Fig. 2).

### Validation of the stability of selected reference genes

**Figure 3:**
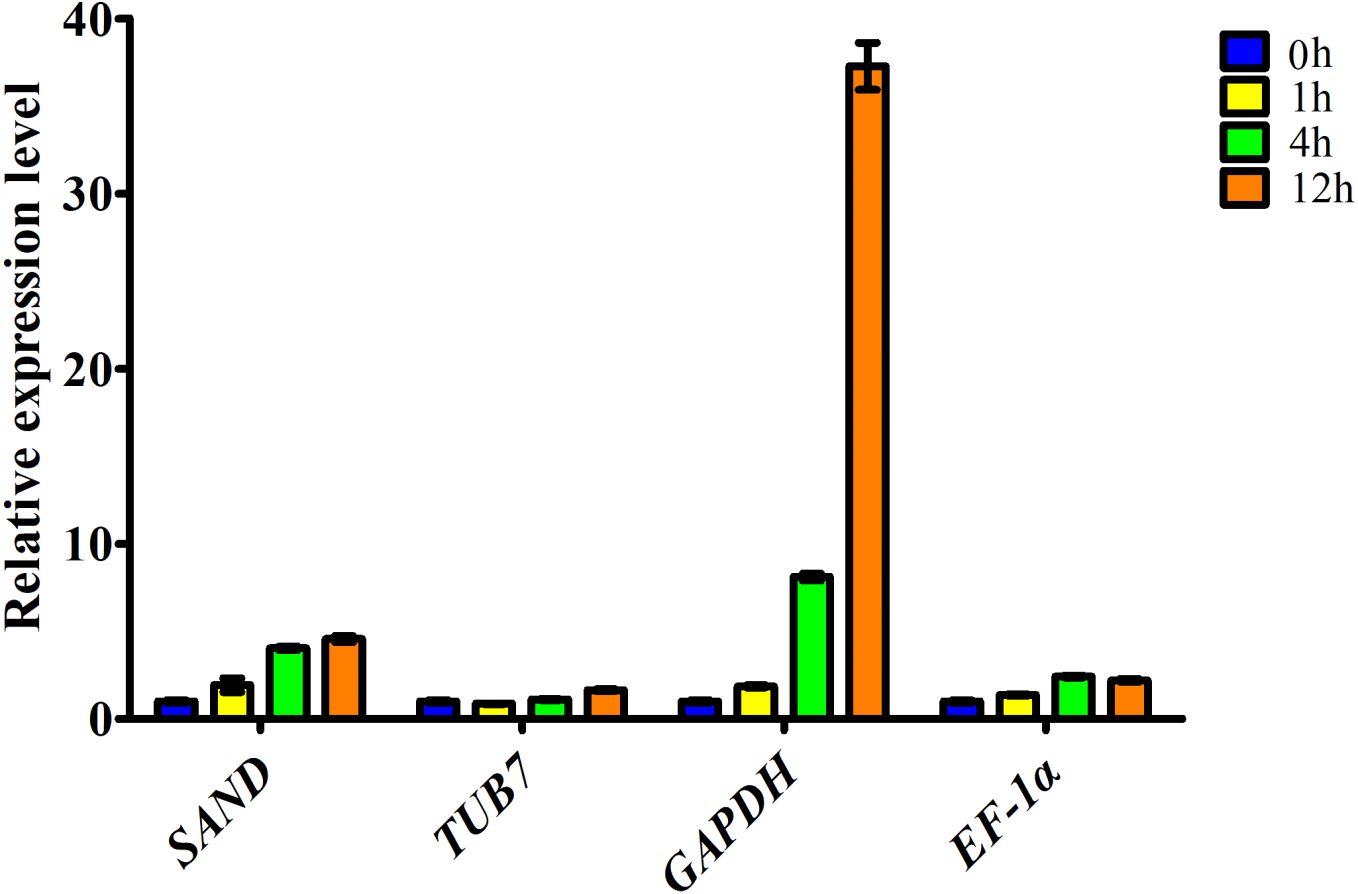
Relative expression level of *ascorbate oxidase* gene in garlic clove exposed to salt treatment for 0, 1, 4, and 12 h. Genes were normalized to *SAND*, *TUB7*, *GAPDH*, and *EF-1α*, respectively. Error bars represent standard deviation from three independent biological replicates.

To determine the accuracy and reliability of candidate reference genes, the expression of *ascorbate oxidase* (*AO*) was calculated with the reference genes selected (Fig. 3). AO is an enzyme involved in the oxidation of ascorbate to monodehydroascorbate using oxygen, and has been documented to be related to salt stress tolerance. Our transcriptome data showed that *AO* can respond to salt stress in garlic cloves, and its expression increased over the period of salinity treatment (Fig. S2).

Here, the expression profiles of *AO* in garlic cloves exposed to salinity were analyzed using four candidate reference genes. When the most stable reference gene in the clove, *SAND*, was used for normalization, transcript levels of *AO* were correlated well with the digital gene expression. By contrast, when the other three less stable reference genes, *TUB7*, *GAPDH*, and *EF-1α* were used, the expression patterns were quite different (Fig. 3).

## Discussion

Despite the fact that powerful modern technology including microarrays and high-throughput sequencing has been utilized to detect gene expression levels, qRT-PCR is still commonly used due to accurate and sensitive quantification of gene expression (Bustin et al., 2005; Nolan, Hands & Bustin, 2006). However, reliable qRT-PCR data are strongly dependent on stable reference genes, which should maintain highly stable expression in distinct biological samples. Comparison and selection of reference genes is a fundamental step for detecting expression variation of target genes and it is essentially required to screen appropriate and stable reference genes for different experimental conditions (Guénin et al., 2009).

Salt stress occurs mainly as a consequence of overuse of chemical fertilizers, industrial waste discharge, and unreasonable irrigation methods (Yu et al., 2016). The soils containing high salt content may result in water stress, imbalance of mineral nutrition and ion absorption, and oxidative damage in plants, thereby, impeding many physiological processes including stomatal aperture, photosynthesis, energy metabolism, and yield formation (Nazar et al., 2011; Xu et al., 2016; Zhu, 2001). Therefore, it is important to identify internal controls for molecular research under salt stress. The expression stability of eight genes in three tissues under salt stress was evaluated with three different software packages, geNorm, NormFinder, and BestKeeper. For the past few years, these statistical algorithms have been introduced to test and identify the most reliable internal controls for qRT-PCR data normalization across a lot of tissues, developmental stages, and adverse circumstances in a variety of plant species, such as brinjal (Kanakachari et al., 2016), pepper (Cheng et al., 2017), persimmon (Wang et al., 2016b), soybean (Wan et al., 2017), and lettuce (Sgamma et al., 2016).

As shown in the box and whisker plot (Fig. 1), the eight genes selected exhibited great variations in expression levels under salt stress, again confirming that no single gene can stay the same expression in any case (Koramutla, Aminedi & Bhattacharya, 2016). Therefore, selection of a most appropriate reference gene is of great importance. As mentioned above, geNorm, NormFinder, and BestKeeper may give different ranking results. For example, *GAPDH* was recognized as the least variable genes in geNorm and NormFinder among the eight genes; however, it was ranked the first place in BestKeeper (Table 2). As a result, different algorithms should be combined to generate the best reference gene (Gimeno et al., 2014). Given all the rankings from the three software packages, we found that *ACTIN* was the best reference gene for normalization in garlic plants under salt stress. *GAPDH* was the least stable and should be avoided as a reference gene in garlic tissues exposed to salinity.

In a previous work, Liu and her colleagues have made great progress in reference gene selection in garlic plants (Liu, Wu & Jiang, 2015). The results indicated that *CYP* was the most suitable reference gene for abiotic stresses. However, the results did not give a specific reference gene just for salt stress. As mentioned above, it is essentially required to select a reliable internal control for a specific condition or stage. Here, *ACTIN* was recognized as the best references gene for salt stress in garlic.

Generally, a cutoff value of 0.15 generated by geNorm is recommended (Vandesompele et al., 2002). If *V* value is below this threshold, there is no need for the introduction of additional reference genes. However, 0.15 is an ideal value that hinges upon the amount of genes and type of tested samples rather than an absolute cutoff value (Hong et al., 2008). In this study, we found that a combination of several reliable reference genes is preferred for gene expression normalization in garlic plants under salt stress. According to geNorm, *V* value showed evident variation at V7/8 in total samples, whereas no evident change was observed in garlic cloves (Fig. 2). The different results may be attributed to the stability (M) of the least stable reference gene calculated by geNorm (Table 2).

*ACTIN* has been commonly used as the internal reference gene in a variety of plant species (Wang et al., 2016a; Xu et al., 2015). However, previous studies also indicated that the expression of this gene may also vary considerably in some cases (Singh et al., 2015). In *Kosteletzkya virginica*, *ACTIN* was not the most reliable gene under salt stress (Tang et al., 2015). However, this gene was proved to be the optimal reference gene across all the samples tested, and performed well in each garlic tissue under salt stress. *SAND* was not an ideal internal control for salt stress in *Oenanthe javanica* (Jiang et al., 2014). By contrast, this gene was suitable for gene expression studies in garlic plants under salt stress. In pigeonpea, *eIF-4α* was not the best reference gene for normalization under salt stress condition (Sinha et al., 2015), which was also detected in the current study. Previous work indicated that the best-ranked reference genes for salt stress were *PP2A* and *TIP41* in *Brassica napus* (Wang et al., 2014). Similarly, in bermudagrass, relatively stable expression was observed for *PP2A* in both roots and leaves subjected to salinity (Chen et al., 2015). By contrast, *PP2A* and *TIP41* should be avoided as a reference gene in the total group because of their poor stability. *TUB7* was suitable for normalizing gene expression in carrot leaves under abiotic stresses (Tian et al., 2015). Here, it was recommended as a suitable reference gene in the total sample pools under salt stress by geNorm and NormFinder. *EF-1α* was recognized as the most appropriate internal controls in *Casuarina equisetifolia* under salt stress (Fan et al., 2017). By contrast, this gene was one of the most variable genes, and should be rejected as reference genes. *GAPDH* is a reliable internal control in both cotton leaves and roots under salt stress (Wang, Wang & Zhang, 2013), whereas maximum variation was detected in transcription data of this gene in the total group.

## Conclusions

In summary, the expression levels of all the eight genes were salt-induced, and it is essential to identify ideal suitable reference genes for garlic plants under salt stress. In the present work, *ACTIN* and *SAND* were the most stable reference genes for salt stress experiments in garlic clove; these two genes also performed well in the total group. In the leaf and root group, *ACTIN* was still the optimal gene for normalizing gene expression. Therefore, these genes are recommended as appropriate reference genes for different garlic tissue types under salt stress. In addition, none of the candidate genes can maintain the same expression in all tissues and conditions. Using a combination of multiple stable reference genes to normalize gene expression is encouraged. The methods and results from the current study can provide aids to the precise quantification of target genes of garlic and other plants.

##  Supplemental Information

10.7717/peerj.7319/supp-1 Table S1 RNA concentration of each sample used for cDNA synthesis

10.7717/peerj.7319/supp-2 Table S2 Nucleotide sequences of the candidate reference genes

10.7717/peerj.7319/supp-3 Table S3 Raw *Ct* values of the eight candidate genes in garlic samples under salt stress

10.7717/peerj.7319/supp-4 Table S4 Primer sequences of ascorbate oxidase for qRT-PCR

10.7717/peerj.7319/supp-5 Figure S1 Standard curves for each of the candidate genes

10.7717/peerj.7319/supp-6 Figure S2 Digital gene expression of ascorbate oxidase in garlic clove under salt stress

## Additional Information and Declarations

### Data Availability

The following information was supplied regarding data availability: The raw measurements are available in the Supplemental Files.
