# Supplementary figures and images for: Selection of reliable reference genes for quantitative RT-PCR in garlic under salt stress
Source: PeerJ. 2019 Jul 16;7:e7319. doi: 10.7717/peerj.7319 (PMC6640627; doi:10.7717/peerj.7319)

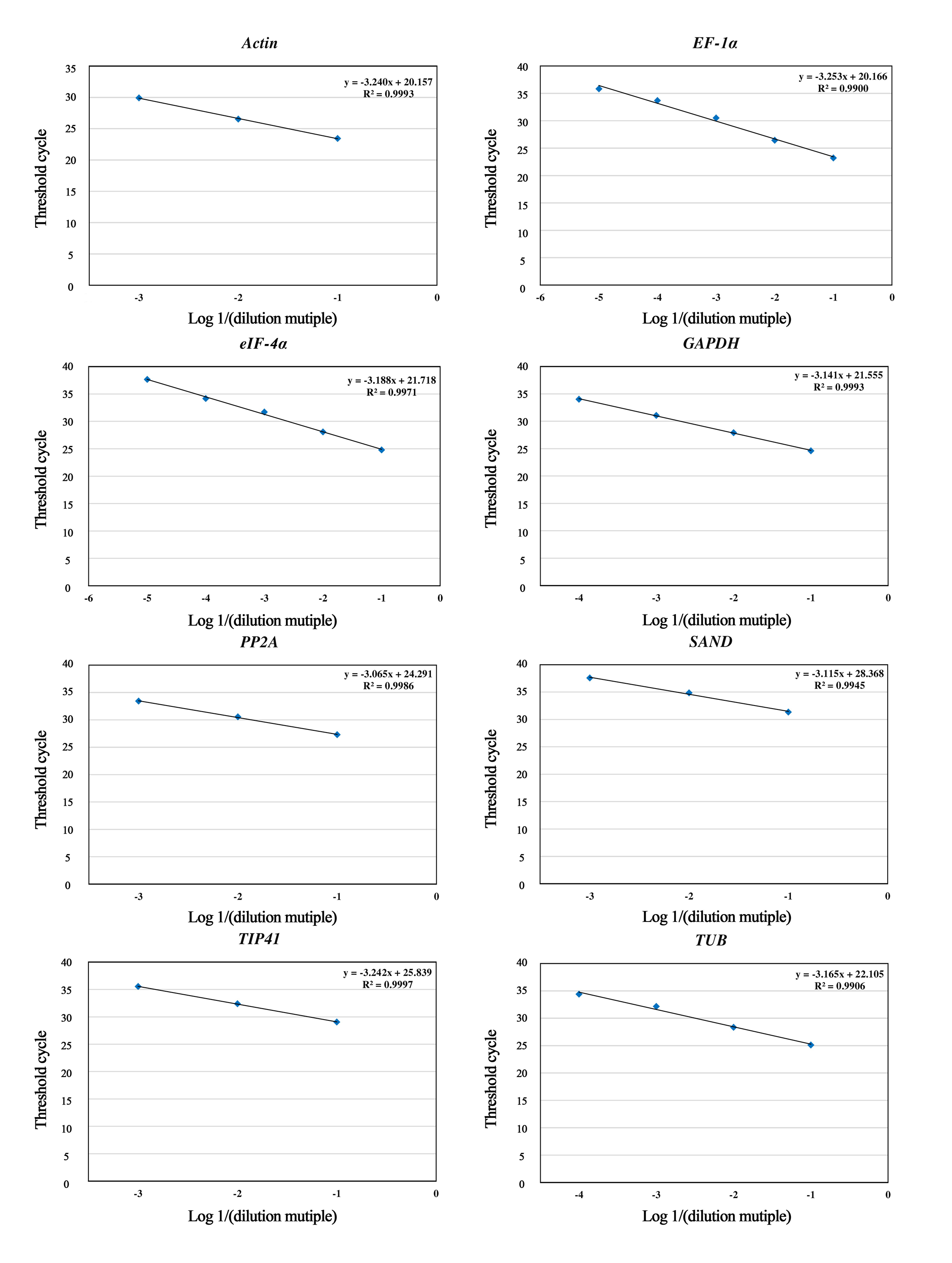

Supplement: Figure S1 [file peerj-07-7319-s005.png]

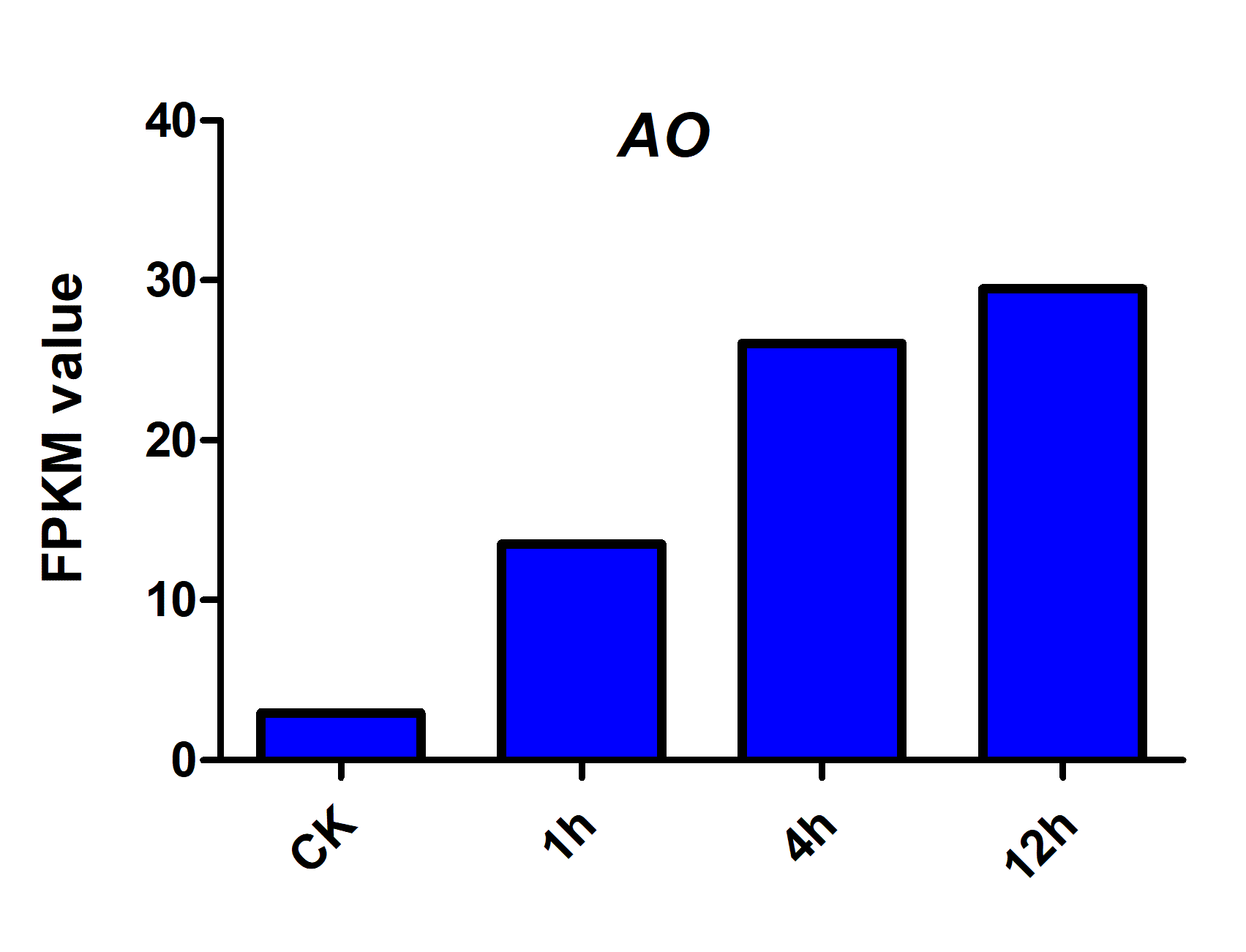

Supplement: Figure S2 [file peerj-07-7319-s006.png]
